# Coenzyme Q10 prevents oxidative stress and fibrosis in isoprenaline induced cardiac remodeling in aged rats

**DOI:** 10.1186/s40360-017-0136-7

**Published:** 2017-04-20

**Authors:** Anayt Ulla, Mustafe Khalid Mohamed, Biswajit Sikder, AFM Towheedur Rahman, Farzana Akther Sumi, Murad Hossain, Hasan Mahmud Reza, G. M. Sayedur Rahman, Md Ashraful Alam

**Affiliations:** Department of Pharmaceutical Sciences, North-South University, Dhaka, 1229 Bangladesh

**Keywords:** Co-enzyme Q10, Mitochondria, Fibrosis, Heart, Oxidative stress

## Abstract

**Background:**

The objective of the present study aimed to investigate the effect of CoQ10 treatment on isoprenaline (ISO)-induced cardiac remodeling in rats.

**Methods:**

Rats were divided into three groups namely Control group, ISO treated group and CoQ10 + ISO treated group, each consisting of 6 rats. The cardiac specific CK-MB, AST, ALT activity and other oxidative stress parameters were estimated in heart and kidneys. Additionally histological examination was also performed to visualize the inflammatory cells infiltration and fibrosis in both tissues.

**Results:**

Administration of ISO resulted in an increase in the heart-to-body weight (HW/BW) ratio and an also increased the serum CK-MB, AST and ALT enzyme activity. Serum levels of lipid peroxidation products, and oxidative stress markers showed significant increase in ISO-treated rats. Histopathological examination of heart tissue revealed focal areas of endocardium degeneration, mononuclear cells infiltration, fibrous tissue deposition, and increased thickness of the myocardium of left ventricle. Similar degeneration was also found in kidneys. Treatment with CoQ10 (100 mg/kg) significantly improved the oxidative stresses in ISO treated rats. Moreover, CoQ10 treatment prevented inflammatory cells infiltration and reduced fibrosis in ISO administered rats.

**Conclusion:**

In conclusion, our study provides evidence that CoQ10 may prevent the development of cardiac remodeling, and fibrosis in ISO administered rats.

## Background

Cardiovascular disease (CVD) became epidemic currently and responsible for the increased mortality and morbidity. At the beginning of the 21^st^ centuries one in every three persons of the world population dies from heart attack, and strokes. Despite all hype about the new drug development, the global cardiovascular epidemic continues relentlessly. WHO information predicted that nearly 23.6 million people will die from CVD each year by 2030 [1]. Cardiac remodeling is usually characterized by expression of genome, changes of molecular, cellular and interstitial state, which ultimately results in changes in size, shape and function of the heart. This is one of the common events that occurred in many cardiovascular disease including myocardial infarction (MI), aortic stenosis, hypertension, myocarditis, idiopathic dilated cardiomyopathy. Cardiac hypertrophy is a typical type of cardiac remodeling [2], where not only the crucial cardiac cell myocyte, but also other components like fibroblasts, collagens, coronary vasculature, interstitium are involved in remodeling process which finally causes ischemia, apoptosis and cell necrosis [3]. Cardiomyocyte hypertrophy is commonly found in aging heart and contributes to the development of congestive heart failure [4]. Stimulation of the sympathetic nervous system is a common attribute in cardiovascular disease [5] and age-related changes in autonomic nervous system function may cause heart-rate variability and increase in resting heart rate. Prolong overstimulation of the β-adrenergic receptor (β-AR) by different stressors causes cardiac hypertrophy, myocardial infarction, strokes, heart failure, coronary artery disease in response to catecholamine and are also responsible for oxidative stress in heart and coronary artery [6]. Auto oxidation of catecholamine generate catecholamine-o-quinones, aminochromes, adrenochrome are thought to be responsible for catecholamine related cardio toxicity [7]. Even previous study have supported that patients with chronic heart failure showed poor survival rate when treated with β-adrenergic agonists [8]. Subcutaneous administration of isoprenaline (ISO), which mimics the β-adrenergic receptor activity, can produce myocardial necrosis [9]. Cardiac hypertrophy induced by isoprenaline is a dependable, consistent and well characterized prototype correlated with arrhythmias, myocyte loss and fibrosis with advancement to heart failure [10].

Oxidative stress and inflammatory responses are one of the underlying mechanisms of myocardial remodeling in aged heart [11]. Excess production of reactive oxygen species and inflammatory cytokines may cause a change in extracellular matrix by activating matrix metalloproteinase (MMP) ultimately results in collagen synthesis and myocardial fibrosis in aged heart [12, 13]. Cardiac apoptosis is another crucial factor to hypertrophic remodeling and cell dysfunction where, ROS stimulate cellular apoptosis signaling kinase-1 and over expression of kinase-1 activates nuclear factor Kappa B (NF-κB) to stimulate hypertrophy [14]. Increasing evidences have suggested that ISO treatment at high doses increases myocardial oxidative stress, pro-inflammatory cytokine synthesis, and stimulate mitogen-activated protein kinases [6, 15]. Functional hypoxia and ischemia, metabolism alterations, coronary insufficiency, energy depletion, calcium overload are some other possible mechanism by which ISO induced cardiotoxicity [16].

Many natural products and dietary supplements containing antioxidant properties demonstrated ROS scavenge in the ISO treated rats [17]. Coenzyme Q10 (Co-Q10), the only synthesized antioxidant in human body is the first drug to improve heart mortality by decreasing all cases of mortality by half [18]. Coenzyme Q10 or ubiquinone is a naturally occurring lipid soluble benzoquinone can be obtained from consumption of meat, poultry, fish, vegetables and fruits [19], a key element for adenosine triphosphate synthesis of the mitochondrial respiratory chain [20]. Co-Q10 can provide protection for membrane phospholipids, mitochondrial membrane protein, and low-density lipoprotein against oxidative damage [21]. Co-Q10 treatment is helpful for lowering pro-inflammatory cytokines and blood viscosity in patients with heart failure and coronary artery disease. It can also improve ischemia and reperfusion injury of coronary revascularization [22]. In addition to its antioxidant activity, Co-Q10 is also responsible for intracellular energy production, responsible for improvement of endothelial dysfunction as well as important for activating mitochondrial uncoupling proteins [23]. Considering the cardioprotective effect of antioxidants, the following investigation was conducted to evaluate the effect of coenzyme Q10 against isoprenaline induced cardiac remodeling in rats.

## Methods

### Chemicals

Co-Q10 was obtained from Medicines Pvt. Ltd. (Dhaka, Bangladesh) and the isoproterenol (ISO) was purchased from Samarth Life Sciences Pvt. Ltd. (Mumbai, India). Thiobarbituric acid (TBA) and 5, 5′-dithiobis-(2-nitrobenzoic acid) (Ellman’s reagent) was purchased from Sigma Chemical Company (USA). Reduced glutathione (GSH) and trichloroacetic acid (TCA) were purchased from J.I. Baker (USA). Alanine aminotransferase (ALT), aspartate aminotransferase (AST), alkaline phosphatase (ALP), uric acid (UA), Creatinin and CK-MB assay kits were obtained from DCI diagnostics (Budapest, Hungary). Sodium hydroxide was collected from Merck (Germany). All other chemicals and reagents used were of analytical grade.

### Animals

Ten to twelve months old, Eighteen Long Evans male rats (235–250 g) were obtained from Animal breeding unit of Animal House at Department of Pharmaceutical Sciences, North South University and were kept in individual cages at room temperature of 22 ± 3 °C and 55% relative humidity with a 12 h dark/light cycles. The animals were provided with standard laboratory chow diet and drinking water *ad libitum*. The whole study protocol to carry out this experiment was approved by Ethical Committee, North South University, Bangladesh for animal care and experimentation.

### Experiment

Eighteen Long Evans aged male rats were evenly divided into 3 groups:

- Group I (*n* = 6)- received olive oil (less than 0.5 ml) and saline (less than 0.5 ml) with normal chow food and water for 14 days.
- Group II (*n* = 6)- Received isoprenaline (ISO) at a dose of 50 mg/kg S.C twice a week for 14 days with olive oil (less than 0.5 ml) and normal chow food and water for 14 days.
- Group III (*n* = 6) – Received CoQ10 100 mg/kg orally in olive oil (less than 0.5 ml) for 14 days daily and ISO 50 mg/kg S.C twice a week for 14 days.

The body weights of all animals were checked and recorded on a daily basis for 14 days. After 14 days of treatment, all the rats were kept at fasting stage for 13–15 h having free access to water only. At the end of the study, all rats were euthanized using high dose pentobarbital sodium (75 mg/kg) and sacrificed. The blood sample was collected from each rat in separate blood collecting tubes pre-coated with anticoagulant citrate buffer. These tubes were then centrifuged at 8000 rpm and separated the plasma. The plasma was stored at −20 °C for further biochemical assays.

Moreover, all organs such as heart, kidney, spleen and liver were also collected immediately after the sacrifice. The collected organs were weighed in an electronic balance and stored both in neutral buffer formalin and at −20 °C for further studies.

### Assessment of cardiotoxicity and kidney function

Several enzymes such as ALT, AST, and ALP activities were determined in plasma by using Diatec diagnostic kits (Hungary) according to the manufacturer’s protocol. The kits for assessing the creatinine kinase-MB (CK-MB), uric acid and creatinin in plasma were also purchased from Diatec diagnostic kits (Hungary) and followed the manufacturer’s standard protocol.

### Preparation of tissue sample for the assessment of oxidative stress markers

To determine the oxidative stress markers, the tissues of heart and kidney were homogenized in 10 volumes of phosphate buffer pH 7.4 and centrifuged at 8000 rpm for 15 min at 4 °C. The upper layer of homogenate (supernatant) was collected and used for the determination of protein and enzymatic studies as mentioned below.

### Estimation of lipid peroxidation as malondialdehyde (MDA), nitric oxide (NO) and advanced oxidation protein products

The concentration of thiobarbituric acid reactive substances (TBARS) in plasma is an index of lipid peroxidation and oxidative stress. Lipid peroxidation in heart and kidney was estimated colorimetrically measuring thiobarbituric acid reactive substances (TBARS) followed by previously described method [24].

NO was determined according to the method described by Tracy et al. as nitrate [25]. NO level was measured by using a standard curve and expressed as nmol/gm of tissue.

A modified method of Witko-Sarsat [26] and Tiwari [27] was performed to determine the AOPP level in plasma and tissue samples. The chloramine-T absorbance at 340 nm being linear within the range of 0 to 100 mmol/mL, AOPP concentrations were expressed as nmol · mL^−1^ chloramine-T equivalents.

### Estimation of catalase activity assay (CAT) and reduced glutathione (GSH) assay

Catalase activity was assayed using a previously described methods by Chance and Maehly [28, 29] with little modifications. One unit of CAT activity was defined as an absorbance change of 0.01 as units/min.

Reduced glutathione was estimated by the method of Jollow et al [30] and expressed as ng/mg protein.

### Histopathological determination

The heart tissues were fixed in 10% neutral buffered formalin and processed using analytical grade ethanol and xylene treatment. The processed tissues were then embedded in paraffin blocks and sectioned to about 5 μm thickness. They were cut by employing a rotary microtome. These sections were stained with Hematoxylin and Eosin using routine procedures. Sirius red staining for fibrosis and Prussian blue staining for iron deposition were also done in heart and kidney sections. Sections were then studied and photographed under light microscope (Zeiss Axio Scope) at 40× magnifications. The slides were examined for morphological changes. Percentage of fibrosis in heart and kidney sections was also analyzed by using Image J free software from National Institute of Health (NIH).

### Statistical analysis

The values are expressed as mean ± standard error mean (SEM). The results were evaluated by using the one way ANOVA followed by Newman Keul’s test using Graph Pad Prism Software. Statistical significance was considered *p* < 0.05 in all cases.

## Results

### Effect of coenzyme Q10 treatment on body weight and organ wet weight in ISO induced rats

On comparing the body weights of three groups, it was observed that the body weight of ISO group and ISO + CoQ10 group did not change significantly compared to control group (Table 1). Furthermore, the ISO treated rats showed significantly increased wet weights of heart, left ventricle and kidney compared to control rats which were ameliorated by CoQ10 treatment. Moreover, ISO treatment did not change the wet weight of spleen among the groups tested in this study.

**Table 1:** Effect of Co-Q10 on body weight, and organ wet weight of ISO treated rats

Parameters	Group		
	Control	ISO	ISO + CoQ10
Initial body weight (g)	234.80 ± 19.31 ns	252.02 ± 6.57 ns	252.56 ± 7.91 ns
Final body Weight (g)	266.53 ± 19.56 ns	249.55 ± 7.91 ns	266.21 ± 9.97 ns
Heart Wet Weight (g/100 g of body weight)	0.26 ± 0.01 ns	0.37 ± 0.01 ns	0.32 ± 0.01 ns
LV(g/100 g of body weight)	0.21 ± 0.01a	0.29 ± 0.01b	0.23 ± 0.01a
RV(g/100 g of body weight)	0.04 ± 0.00 ns	0.05 ± 0.01 ns	0.05 ± 0.01 ns
Kidney(g/100 g of body weight)	0.54 ± 0.02a	0.64 ± 0.02b	0.55 ± 0.02a
Spleen(g/100 g of body weight)	0.33 ± 0.02 ns	0.34 ± 0.01 ns	0.30 ± 0.01 ns

Data are presented as mean ± SEM, n = 6. Statistical analysis was done as One way ANOVA with Newman Keuls test as *post hoc* test, which was conducted using Prism software (USA). a vs b is significantly different at *p* < 0.05. a vs b, control vs ISO or ISO vs ISO + CoQ10. Others are not significant

### Effect of coenzyme Q10 on AST, ALT and ALP activity

ISO administration in rats considerably increased the AST, ALT and ALP enzymes activities compared to the control rats (Table 2). CoQ10 administration significantly (*p* < 0.05) lowered the AST and ALP activities but no such change was seen for ALT activity (Table 2).

**Table 2:** Effect of Co-Q10 on biochemical parameters in ISO induced aged rats

Parameters	Group		
	Control	ISO	ISO + CoQ10
Plasma			
AST (U/L)	29.29 ± 4.39a	51.68 ± 4.72b	33.02 ± 3.46a
ALT (U/L)	33.02 ± 4.67a	78.96 ± 18.79b	44.50 ± 6.06a
ALP (U/L)	57.60 ± 4.88a	82.15 ± 5.85b	60.67 ± 3.11a
MDA (nmol/mL)	78.51 ± 6.12a	174.54 ± 6.11b	101.72 ± 10.89a
NO (nmol/mL)	3.82 ± 0.25a	11.12 ± 1.69b	6.82 ± 0.55a
APOP (nmol/mL equivalent to Chloramine-T)	229.13 ± 10.64a	382.30 ± 31.75b	200.16 ± 19.43a
Catalase (U/min)	22.50 ± 4.23a	10.00 ± 1.83b	15.00 ± 2.58a
GSH (ng/mg protein)	11.59 ± 0.46 ns	7.16 ± 0.53 ns	13.65 ± 0.53 ns
CK-MB (U/L)	136.11 ± 19.44a	299.44 ± 31.55b	151.67 ± 34.48a
Uric Acid (μmol/L)	5.02 ± 0.52a	10.65 ± 0.75b	7.82 ± 0.56b
Creatinin (μmol/L)	1.36 ± 0.10a	2.34 ± 0.19b	1.52 ± 0.03b
Heart			
MDA (nmol/gm tissue)	52.62 ± 3.96a	102.62 ± 4.40b	80.56 ± 6.10a
NO (nmol/gm tissue)	13.26 ± 0.64 ns	15.49 ± 1.06 ns	8.38 ± 0.28 ns
APOP (nmol/gm tissue equivalent to Chloramine-T)	624.13 ± 29.80a	924.92 ± 107.66b	569.37 ± 38.78a
Catalase (U/min)	145.83 ± 18.68a	83.33 ± 8.82b	94.17 ± 6.38a
GSH (ng/mg protein)	12.47 ± 0.44 ns	9.38 ± 0.81 ns	16.77 ± 1.04 ns
Kidney			
MDA (nmol/gm tissue)	40.31 ± 0.79a	85.82 ± 2.55b	66.85 ± 2.01b
NO (nmol/gm tissue)	21.54 ± 1.43a	33.82 ± 2.38b	18.77 ± 2.30a
APOP (nmol/gm tissue equivalent to Chloramine-T)	711.43 ± 20.75a	1143.97 ± 96.08b	911.43 ± 68.81b
Catalase (U/min)	36.67 ± 5.11a	16.67 ± 2.47b	29.17 ± 2.71a
GSH (ng/mg protein)	17.94 ± 0.42 ns	12.84 ± 0.57 ns	15.05 ± 2.42 ns
Urine			
	Control	ISO	ISO + COQ10
Uric Acid (μmol/L)	6.02 ± 0.06a	9.42 ± 0.26b	7.13 ± 0.25a
Creatinin (μmol/L)	8.16 ± 0.75a	17.75 ± 2.32b	7.03 ± 1.18a

Data are presented as mean ± SEM, *n* = 6. Statistical analysis was done as One way ANOVA with Newman Keuls test as *post hoc* test, which was conducted using Prism software (USA).). a vs b is significantly different at *p* < 0.05. a vs b, control vs ISO or ISO vs ISO + CoQ10. Others are not significant

### Effect of coenzyme Q10 treatment on CK-MB, uric acid and creatinin activities in ISO induced rats

ISO administered rats showed significant (*p* < 0.05) higher activity of CK-MB in plasma compared to control rats. CoQ10 administration significantly (*p* < 0.05) lowered the CK-MB activity in ISO administered rats (Table 2).

ISO administered rats also showed significant (*p* < 0.05) higher level of uric acid and creatinin level in plasma compared to the control rats. Similar result was also found for uric acid and creatinin on analyzing the urine sample. Interestingly, the CoQ10 treatment significantly (*p* < 0.05) reduced the level of uric acid and creatinin concentration in urine sample but not in plasma sample in ISO administered rats.

### Effect of coenzyme Q10 treatment on oxidative stress parameters and antioxidant enzymes on ISO induced rats

To study the oxidative stress and antioxidant parameters, malondialdehyde (MDA), nitric oxide (NO), advanced protein oxidation product (APOP), catalase (CAT) and glutathione (GSH) levels in plasma and tissue samples were analyzed. ISO treatment in rats showed an increased level of lipid peroxidation product MDA in plasma and tissues compared to the control rats (Table 2). ISO treatment also raised the nitric oxide and advanced protein oxidation product in plasma and tissues compared to control rats. However, antioxidant enzymes CAT activity and GSH concentration were decreased in plasma and tissues of ISO treated rats compared to control rats. Coenzyme Q10 treatment prevented the rise of lipid peroxidation product MDA, NO, and APOP concentration significantly in both plasma and tissues (Table 2). Moreover, CoQ10 treatment effectively restored the GSH level in ISO administered rats (except in kidney tissue homogenates) but the catalase activity was not restored significantly by CoQ10 treatment.

### Effect of coenzyme Q10 treatment on histological assessments in heart and kidney structure in ISO induced rats

**Fig. 1:**
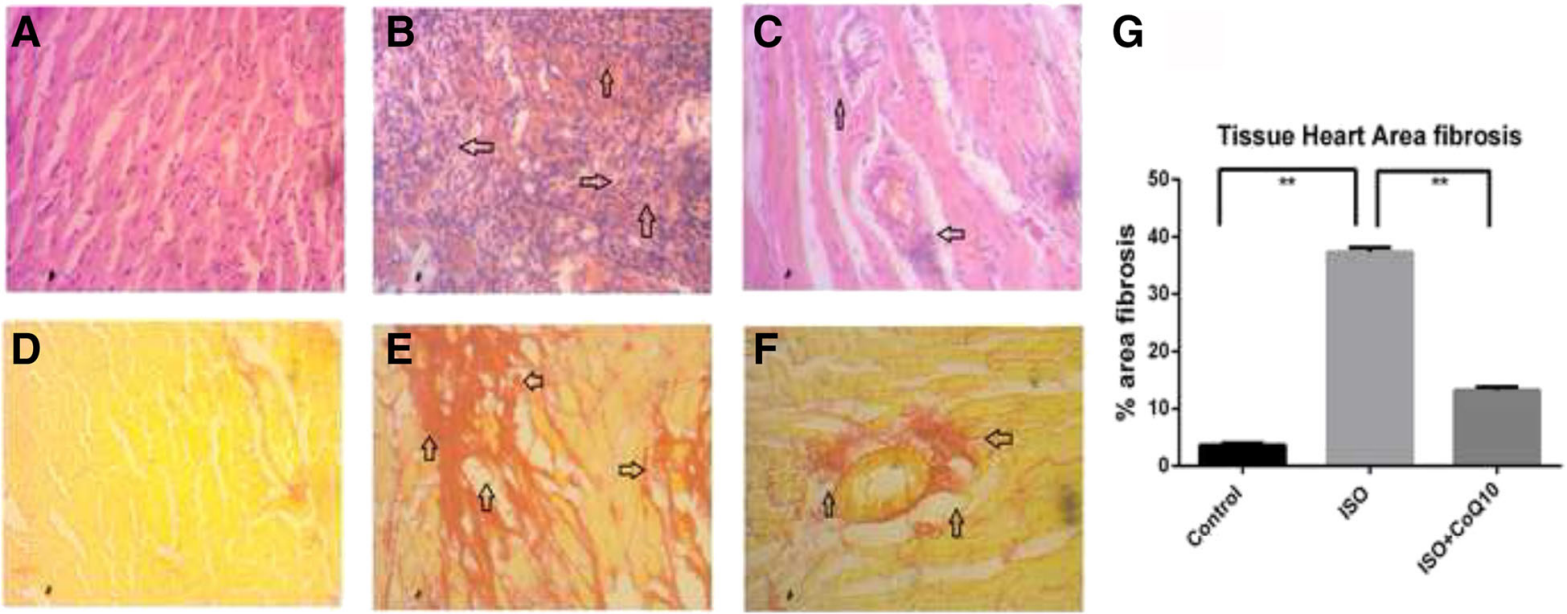
Effect of Co-Q10 treatment on cardiac inflammation (*Upper Panel*, *arrow head*- inflammatory cells infiltration) and fibrosis (*Lower panel, arrow head*- collagen deposition) in ISO administered rats. **a**, **d**- Control; **b**, **e**- ISO; **c**, **f**- ISO + CO-Q10. **g**, % of fibrosis among groups. Magnification 40 X. Asterisk mark is significantly different at *p* < 0.05. Control vs ISO or ISO vs ISO + CoQ10. Others are not significant

Mononuclear inflammatory cells infiltration in heart was observed in ISO treated group compared to control group (Fig. 1). CoQ10 treatment averted the inflammatory cells infiltration in heart of ISO administered rats. Besides that, ISO administered rats showed hypertrophy of cardiomyocytes and massive fibrosis along with inflammation (Fig. 1). Treatment with CoQ10 was also helpful for ameliorating fibrosis in ISO administered rats (Fig. 1).

**Fig. 2:**
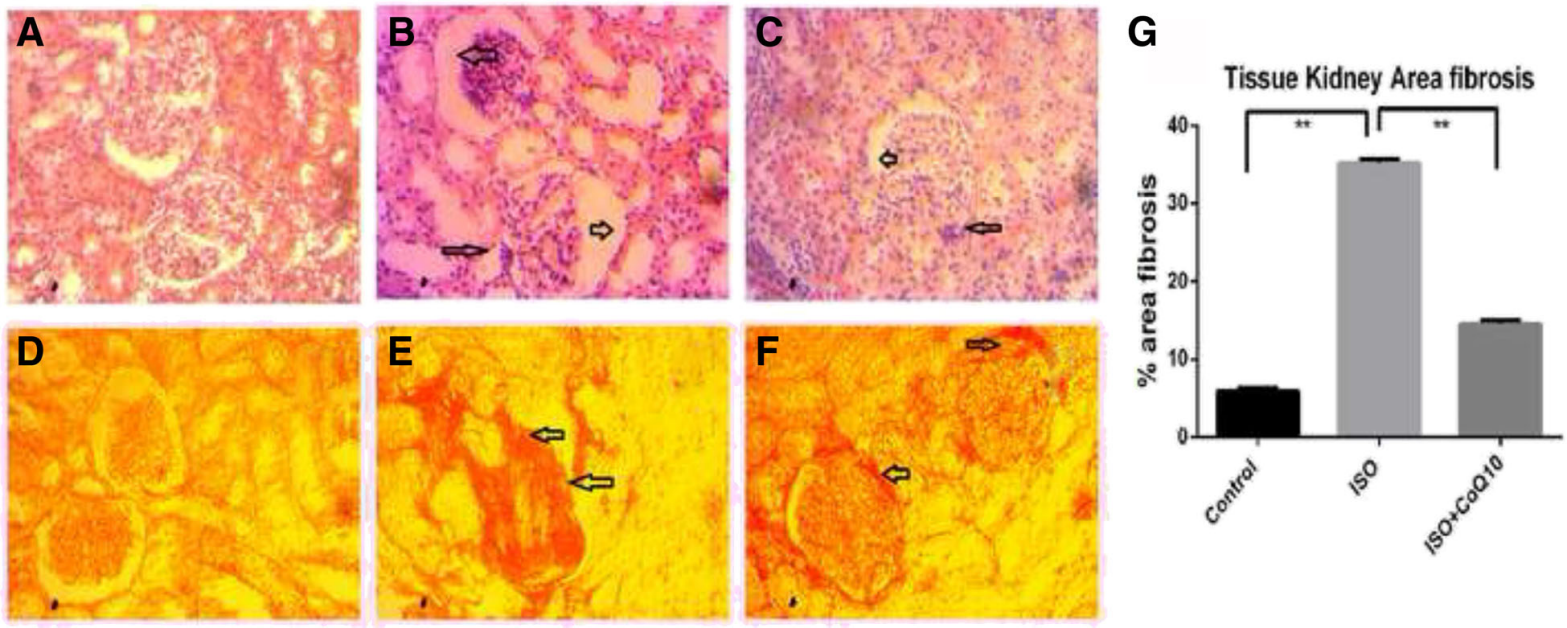
Effect of Co-Q10 treatment on glomerular structure (*Upper Panel, arrow head*- podycyte damage, shrinkage of glomerulus) and fibrosis (*Lower panel, arrow head*- collagen deposition) in kidneys of ISO administered rats. **a**, **d**- Control; **b**, **e**- ISO; **c**, **f**- ISO + CO-Q10. **g**, % of fibrosis among groups. Magnification 40 X. Asterisk mark is significantly different at *p* < 0.05. Control vs ISO or ISO vs ISO + CoQ10. Others are not significant

**Fig. 3:**
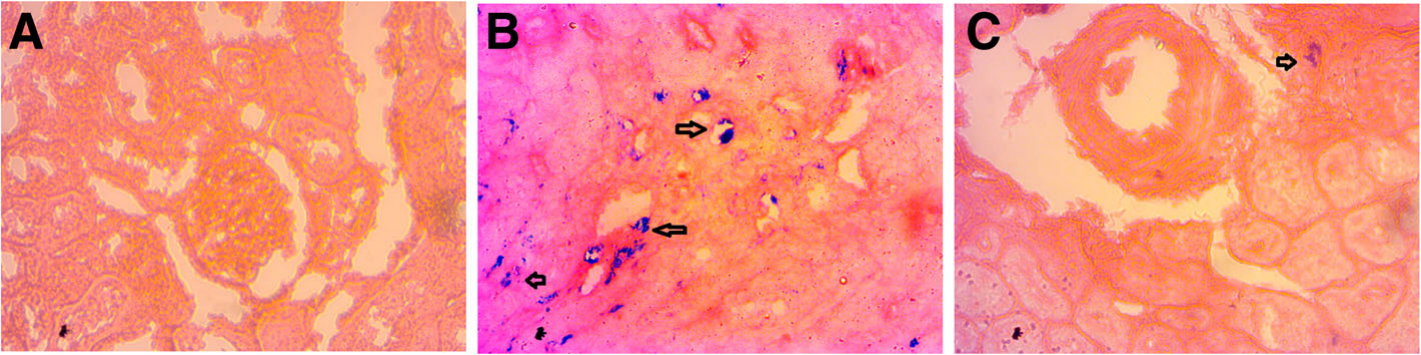
Effect of Co-Q10 treatment on iron deposition (*Arrow head*) in kidneys of ISO administered rats. **a**- Control; **b**- ISO; **c**- ISO + CO-Q10. Magnification 40 X

Histopathological analysis of kidney sections obtained from control rats showed normal architecture of the kidney. It was devoid of congestion, necrosis, fibrosis and inflammatory infiltration (Fig. 2). Basement membrane of the glomerulus was remained intact. ISO treatment in rats also changes the function and structure of kidneys. Kidney form ISO treated rats showed renal damage evident by glomerular structural disruption and fibrosis. It also showed the presence of intraluminal cell debris, edema and inflammatory cells infiltration (Fig. 2). Moreover, ISO treated rats showed iron deposition in kidney sections which may be due to oxidative stress (Fig. 3). Co-Q10 treatment in ISO administered rats improves the kidney structure (Fig. 2) by lowering inflammatory cells infiltration and fibrosis. Co-Q10 treatment in ISO administered rats also prevented the deposition of free iron in kidney sections (Fig. 3).

## Discussion

Aging is a retrogressive process that is kindred with ongoing accumulation of detrimental changes with time, results in reduction of physiological such as cardiovascular, renal, neurological, endocrine function with the increase of chances of disease and death. With advancing age one of the most debilitating is loss of myocardial function. Decreased cardiac elasticity and inability to respond of changes in pressure to the arterial system mainly influence the heart function associated with aging [31]. Cardiac tissue is mainly postmiotic and oxidative damage is most pervasive because of its high dependency on oxidative phosphorylation to derive energy [32]. Altered inflammatory mediator expression and inability to respond of senescent cells to growth factors are important mechanism of age related adverse cardiac remodeling [33]. Besides, in aging and ischemic disease, ROS are responsible for peroxidation of mitochondrial phospholipids. Mitochondrial respiratory chain is mainly responsible for production of these ROS free radicals [34]. In this study, cardiac remodeling in rats was induced by subcutaneous injection of isoprenaline. At low doses, catecholamine exerts positive inotropic effect that is beneficial for heart function. But high doses of isoprenaline causes energy depletion of heart resulting in biochemical and structural changes of cardiomyocyte [16]. This study showed that isoprenaline induced cardiac hypertrophy by increasing left ventricular wet weight. Isoprenaline administration also raised the level of different oxidative stress markers and lowered the level of antioxidant from plasma and heart. Colossal amount of infiltrating cell and fibrosis was found on infract area induced by isoprenaline. In mitochondria, 80% oxygen consumption is occurred and this very compartment is mainly responsible for production of O_2_^.-^ and H_2_O_2_ [23]. The mitochondrial element coenzyme Q10 (Co-Q10) or ubiquinone has been used as a dietary supplement for improving health condition by eradicating free radicals [32]. Several previous studies have revealed the beneficial effect of CoQ10 in aging [35–37]. Previous studies showed that CoQ10 level was found low in patients with cardiovascular disease [18, 19]. Generally, CoQ10 or ubiquinone in its reduced form acts as an antioxidant and lowers different inflammatory markers like TNF-α and IL-6 [38]. This research suggests that treatment with CoQ10 helps to minimize oxidative stress, inflammatory cell infiltration and fibrosis in heart in isoprenaline treated rats.

Damage to cardiac tissue induced by different β-adrenergic receptor agonist like isoprenaline increases the level of ALT, AST, and ALP enzymes [39]. Myocardial necrosis induced by ISO treatment increases ALT, AST and ALP activities are observed in plasma. In our study, CoQ10 significantly inhibit the increase of AST and ALP. Previous study also supported that treatment with CoQ10 can reduce these enzyme activities from plasma [40]. Creatine kinase-MB (CK-MB) is a superior and special marker, serves as an indicator of myocardial necrosis [41]. Elevation of CK-MB activity because of ISO treatment is observed in plasma of aged rats [42]. In this study, treatment with CoQ10 inhibited the elevation of CK-MB activity in plasma. This study is also supported by previous finding which also showed CoQ10 can reduce CK-MB activity in isoprenaline induced cardiotoxicity and cardiac hypertrophy in rats [43].

Oxidative stress emanating from subcutaneously isoprenaline injection is mediated mainly through β-adrenergic receptor stimulation which rapidly generates ROS by spontaneous oxidation of catecholamine. Isoprenaline administration results in increase calcium overload and myocardial dysfunction in cardiomyocytes [44]. Increased lipid peroxidation is an important biomarker indicative of elevated oxidative stress. Alterations of balance between prooxidant and antioxidant defense is thought to be the main mechanism behind lipid peroxidation where, ATP is converted to hypoxanthine and uric acid respectively [45]. Myocardial phospholipids membrane peroxidation causes permeability change and intracellular calcium overload results in cardiomyocytes damage in the heart [46]. Catecholamine induced increase in MDA level in myocardial tissue was reported in previous study [47]. Increased malondialdehyde (MDA, product of lipid peroxidation) content was increased in ISO treated rats that were attenuated by CoQ10 in our study. Besides, another novel oxidative stress marker AOPP (Advanced Oxidation Protein Products) level was high in ISO treated group which was lowered significantly by CoQ10 treatment. Nitric oxide is an another reactive molecule that can control cardiovascular homeostasis, myocyte growth, function and remodeling [14]. Uncoupled NO-synthase is the main contributor of ROS generation which ultimately results in endothelial dysfunction [48]. In cardiac hypertrophy induced by isoprenaline, increased iNOS expression in left ventricle and increased level of NO production in heart was reported associated with increased apoptosis [10]. Mainly, activation of β-adrenergic receptor is the main mechanism of up regulation of iNOS [49]. This excess nitric oxide concentration can react with superoxide radical and can form peroxynitrite (˙ONOO-) a highly toxic reactive species capable of activating cytotoxic process including, protein oxidation, lipid peroxidation and nitration ultimately results in myocardial injury [14]. In this study, nitric oxide level was also found to be elevated in ISO treated rats whereas, mitochondrial component CoQ10 successfully normalized the elevation of nitric oxide level. Increased heart wet weight was observed in ISO treatment which was normalized by CoQ10 treatment. Indeed, Akt signaling induces physiological hypertrophic responses important for controlling cardiac structure and function. Previous study proved that cardioprotective effect of CoQ10 lies on preservation of Akt signaling where coenzymeQ10 can protect mice from pathological cardiac hypertrophy [50]. CoQ10 can efficaciously reduce alpha-tocopheroxyl radical to alpha-tocopherol. Thus, having the property of terminating pro-oxidant radical, CoQ10 can regenerate the active form of vitamin E [34].

Antioxidant contrives the defense mechanism capable of decelerating or averting the unstable free radicals from initiating toxic effects that simultaneously causes tissue damage. The prime endogenous antioxidants such as SOD (superoxide dismutase), CAT (catalase), GSH (reduced glutathione) are responsible for trapping free radicals where, SOD catalyses the conversion of O_2_^.-^ to H_2_O_2_ and H_2_O; CAT converts H_2_O_2_ to H_2_O and O_2_; glutathione reduces H_2_O_2_ to H_2_O. Myocardial injury induced by ISO treatment increases lipid per oxidation which in turn reduces these enzyme levels [51, 52]. This study also showed significant reduction of CAT activity and GSH level in ISO treated rat both in plasma and heart compared to control group. CoQ10 treatment restored the GSH level significantly.

ISO is considered as a model compound that causes cardiac inflammation and fibrosis. It has been postulate that this beta adrenergic agonist results in hypoxic condition in cardiac tissue as a result of uncontrolled oxidative metabolism in myocyte. The imbalance of energy, in addition with a number of complex biochemical (altered calcium flux, stimulation of the adenyl cyclase system, aggregation of platelets, and formation of reactive oxygen species) [53] and structural changes (alterations in membrane permeability) [54] & [55], appear to contribute to the pathogenesis of the myocyte damage [56]. The area of the heart most susceptible to hypoxia caused by tachycardia appears to be the left ventricular subendocardium [53]. Recently, changes in iNOS expression have also been associated with ISO-induced cardiotoxicity [57]. Myocyte damage observed following exposure to ISO includes both apoptosis and necrosis [58]. Cardiac inflammation by ISO was observed by histological evaluation which demonstrated significant endomyocardial eosinophilic infiltration and areas of myocyte necrosis [59]. On the other hand myocardial fibrosis was identified by the appearance of thick, coarse groups of interstitial collagen fibrils associated with a few necrotic myocytes [60]. This study showed that administering ISO resulted in the massive mono nuclear cells infiltration as well as deposition of interstitial collagen fibrils on histological analysis of cardiac and kidney tissues which strongly supported by previous findings [61]. Both scenarios were reversed after the treatment with CoQ10.

Kidney dysfunction may also contribute to the development of cardio-vascular complications [61, 62]. Isoprenaline induced renal complications and fibrosis was also reported in animal studies. Recent evidence also suggests that ISO administration increase cardio-renal fibrosis which was ameliorated by sympathetic nerve system (SNS) inhibition by denervation procedure [62]. This study suggests that Co-Q10 administration prevents inflammation and fibrosis in kidney of ISO treated rats. Improved cardiac and kidney tissues structure due to Co-Q10 administration could be relied on the improvement of oxidative stress and restoration of antioxidant defence in tissues which is also observed in other studies as well [63, 64].

## Conclusion

The results of this study indicate that the coenzyme Q10 treatment provides significant cardiac and renoprotective effect against ISO administered rats. Coenzyme Q10 inhibits cardio-renal fibrogenesis through reducing oxidative stress and preventing inflammatory cells infiltration in tissues of ISO treated rats. Thus targeting mitochondrial function would be a possible way of preventing oxidative stress related disorders in future.

## Abbreviations

ALP: Alkaline phosphatase
ALT: Alanine aminotransferase
APOP: Advanced protein oxidation product
AST: Aspartate aminotransferase
CAT: Catalase
CK-MB: Creatinin kinase muscle-brain
Co-Q10: Coenzyme Q10
CVD: Cardiovascular disease
DTNB: 5, 5′-dithiobis-(2-nitrobenzoic acid)
GSH: Reduced glutathione
H_2_O_2_: Hydrogen per Oxide
iNOS: Inducible nitric oxide synthase
ISO: Isoprenaline
MI: Myocardial infarction
MMP: Matrix metalloproteinase
NF-κB: Nuclear factor Kappa B
NO: Nitric oxide
ROS: Reactive oxygen species
SOD: Super oxide dismutase
TBA: Thiobarbituric acid
TBARS: Thiobarbituric acid reactive substances
TCA: Trichloroacetic acid, ˙ONOO, Peroxynitrite
UA: Uric acid
